# The Effect of Diet and Opponent Size on Aggressive Interactions Involving Caribbean Crazy Ants (*Nylanderia fulva*)

**DOI:** 10.1371/journal.pone.0066912

**Published:** 2013-06-11

**Authors:** Katherine C. Horn, Micky D. Eubanks, Evan Siemann

**Affiliations:** 1 Department of Ecology and Evolutionary Biology, Rice University, Houston, Texas, United States of America; 2 Department of Entomology, Texas A&M University, College Station, Texas, United States of America; University of Sussex, United Kingdom

## Abstract

Biotic interactions are often important in the establishment and spread of invasive species. In particular, competition between introduced and native species can strongly influence the distribution and spread of exotic species and in some cases competition among introduced species can be important. The Caribbean crazy ant, *Nylanderia fulva*, was recently introduced to the Gulf Coast of Texas, and appears to be spreading inland. It has been hypothesized that competition with the red imported fire ant, *Solenopsis invicta*, may be an important factor in the spread of crazy ants. We investigated the potential of interspecific competition among these two introduced ants by measuring interspecific aggression between Caribbean crazy ant workers and workers of *Solenopsis invicta*. Specifically, we examined the effect of body size and diet on individual-level aggressive interactions among crazy ant workers and fire ants. We found that differences in diet did not alter interactions between crazy ant workers from different nests, but carbohydrate level did play an important role in antagonistic interactions with fire ants: crazy ants on low sugar diets were more aggressive and less likely to be killed in aggressive encounters with fire ants. We found that large fire ants engaged in fewer fights with crazy ants than small fire ants, but fire ant size affected neither fire ant nor crazy ant mortality. Overall, crazy ants experienced higher mortality than fire ants after aggressive encounters. Our findings suggest that fire ant workers might outcompete crazy ant workers on an individual level, providing some biotic resistance to crazy ant range expansion. However, this resistance may be overcome by crazy ants that have a restricted sugar intake, which may occur when crazy ants are excluded from resources by fire ants.

## Introduction

Biotic interactions between introduced species and native or pre-established exotic species are important influences on the success and spread of invasive species [1]–[7]. Competition from established species in the introduced range can sometimes serve as biotic resistance to invasive species [8], [9]. Competitive interactions can be exploitative, where individuals compete indirectly via their effects on shared resources, or interference, where individuals directly clash through antagonistic behaviors [10]. In ants, interference competition is common and can cause death or injury of workers and loss of access to food or territory [11]–[13]. In this study, we explored the potential for competition among two invasive ants by quantifying the aggressive interactions and resulting mortality among workers of the recently introduced Caribbean crazy ant, *Nylanderia fulva* (Hymenoptera, Formicidae, Formicinae), and the established invasive red imported fire ant *Solenopsis invicta* (Hymenoptera, Formicidae, Myrmicinae).

Aggressive interactions among ants, however, can be mediated by diet. The diet of an ant can influence aggressive interactions between species or colonies of a single species via two mechanisms. First, the cuticular hydrocarbons of prey items can be transferred to foraging ants, altering the ant’s hydrocarbon profile and increasing aggression between nestmates [14]–[16] and between nests or colonies of a given species [17]–[19]. Second, some ants are more aggressive when they consume a diet rich in carbohydrates [20]. For example, the amount of sugar in a colony’s diet has been shown to be positively correlated with aggressive behavior in both *Formica aquilonia* and invasive Argentine ants, *Linepithema humile* [19], [16]. Because of the importance of diet in interactions between ants, we examined the effects of both sugar level and prey type on intra- and interspecific aggression in Caribbean crazy ants.

*Nylanderia fulva* was first discovered in Texas in an industrial area along the ship channel in Pasadena, TX in 2002 [21]. It was originally described in Texas as *Paratrechina* sp. nr. *pubens* and given the common name “Rasberry crazy ant” [21]. Subsequently, it has been shown that *N. pubens* (Caribbean crazy ant – present in Florida for 60 years [22]) and *P.* nr. *pubens* are the same species [23] and that they are in fact *N. fulva* [24]. Since its introduction to Texas, the range of Caribbean crazy ants has increased by 20–30 m per month [21]. Media reports on the ant focus on the tendency of Caribbean crazy ants to nest in electronics and cause short circuits. Though these effects of the crazy ants are likely overstated, it is known that Caribbean crazy ants are often found in extremely high densities in invaded areas [21]. Crazy ant populations appear to be unicolonial; colony boundaries seem to be nonexistent as ants move freely between nests [12]. Crazy ants are often found in areas that would be suitable habitat for red imported fire ants [23], such as woods and open areas, suggesting that the two species may often come into contact and compete for resources.

Red imported fire ants are themselves one of the world’s top 100 worst invasive species [25]. Introduced to the United States via the port city of Mobile, Alabama in the 1930’s [26], fire ants have since spread throughout more than 106 million hectares of the southeastern US, the Midwest, and California, where they are the dominant ant species in disturbed habitats [27]. They are size polymorphic and also form super-colonies. Due to their harmful effects on humans, agriculture, and ecosystems, fire ants cost nearly one billion dollars per year in economic losses and control efforts [28]. Some news reports have suggested that crazy ants attack, eat, and displace fire ants, yet none of these claims have been tested. Due to the widespread invasion of fire ants and the locally abundant populations of Caribbean crazy ants, interactions between these two species may be very important in affecting the spread of crazy ant populations.

To examine the intra- and interspecific interactions of the Caribbean crazy ants, the following sets of ant pairings were observed for aggression: 1) crazy ants from nests in the same supercolony which had been isolated and fed one of two prey types and either high or low doses of sugar water, 2) crazy ants which had been fed the different diets described above together with fire ants, and 3) crazy ants and either small or large fire ant workers. All interactions were examined using aggression assays with five ants from each species or treatment in a Petri dish. Aggression assays have been shown to be highly consistent and correlate well with full colony introductions in a number of ant species [29]. Together, these three sets of aggression assays made it possible to address the following questions: 1) Do differences in diet affect interactions between workers of different crazy ant nests? 2) Are crazy ants or fire ants more successful in fights? 3) Can the diet of crazy ants affect aggressive interactions with fire ants? 4) Is fighting success of crazy ants affected by fire ant size?

## Methods

### Nest Establishment

All nests of Caribbean crazy ants used in aggression assays were collected in a public right-of-way in Pearland, TX (29.55°N, 95.28°W) on May 24 and May 31, 2008. No permission was required to collect in this area and crazy ants are not an endangered or protected species. Though 24 distinct nests were collected, because crazy ants at this site display no aggression among intraspecifics, it is likely that all nests are parts of a large supercolony [12]. Fire ant nests were collected at Katy Prairie Conservancy in Katy, TX (29.93°N, 95.94°W) in early May 2008. Nests were collected by digging up them up and transferring them to buckets. Nests were kept separate. We received permission to collect in this area and fire ants are not an endangered or protected species. In order to separate ants from the nesting material with which they were collected, we flooded nests and then transferred all ants to 24 cm×11 cm plastic nest boxes. Nest boxes were ringed with a thin layer of Tanglefoot (Tanglefoot, Grand Rapids, Michigan, USA) near the top of the inside walls in order to prevent escapes. These nest boxes were then placed in larger containment vessels that were set-up such that ants were contained by two moats of soapy water, a layer of baby powder, and two additional rings of Tanglefoot.

The initial diet administered to both crazy ant and fire ant nests consisted of freezer-killed mealworms and sugar water (4.2%). This diet was maintained until July 18, 2008, when diet manipulations began. All food was removed from crazy ant nest boxes, and each of the 24 crazy ant nests was assigned to one of four treatments: cricket/high sugar, cricket/low sugar, wax worm/high sugar, or wax worm/low sugar, such that there were six nests in each treatment. The low concentration sugar water consisted of 5 ml of sugar in 120 ml of water (4.2% sugar by volume); the high concentration sugar water consisted of 20 ml of sugar in 120 ml of water (16.7% sugar by volume). Each colony was given half of a freezer-killed cricket (Orthoptera, Gryllidae) or half of a freezer-killed mealworm and 7.4 ml of sugar water of the appropriate concentration every other day. Wax worms (Lepidoptera, Pyralidae) were supplied by Armstrong’s Cricket Farm (West Monroe, LA) and crickets were purchased at a local pet supply store.

### Aggression Assays

The following aggression assays were performed: five crazy ant workers in a Petri dish with either five small or five large fire ant workers, five crazy ant workers from one diet treatment in a Petri dish with five crazy ants from a colony receiving a different diet treatment, and five small fire ants with five crazy ants on an experimental diet. For each aggression assay, ants were placed in a 9-cm Petri dish with Fluon (polytetrafluoro-ethylene)-coated sides that prevented ant escape during these periods of observation. The Suarez scale [30] was then used to score the behavior of pairs of interacting ants every minute for either five or ten minutes (see below), depending on the species combination: 0– ants had no interaction (i.e. ignored each other), 1 - attenuation was observed, 2 - avoidance, 3– aggression (such as biting antennae or legs), and 4– fighting (both ants engaged). The number of ants from each treatment or colony engaged in fights was also recorded each minute. We did not attempt to distinguish which individual ants were involved in interactions. In all combinations, the first observation was made five seconds after all ants were released into the Petri dish. At the end of the 5- or 10-minute observation period, the ants were then left in the Petri dish for one or two hours, and mortality was recorded after each hour. Individuals were only used in a single trial. All statistical analyses were performed using JMP 7.0.2 (SAS Institute Inc.). Each of the aggression assays is explained in further detail below.

### Crazy Ants Receiving Different Diets

Intraspecific aggression trials were conducted on July 29, 2008. A total of 24 pairings of nests were used. Some source nests were used more than once, but all pairings were unique. Of these 24 pairings, eight differed by prey type, six differed by sugar level, six differed by both factors, and four were pairings of nests from the same treatment. In order to distinguish between the two treatments of crazy ants in aggression trials, workers were coated with either pink or green fluorescent powder (Day Glo Color Corp. Cleveland, Ohio, USA) using a small paintbrush in an intermediate holding container a few minutes before they were added to the Petri dish. After the ants had ceased grooming and resumed moving about the container, they were placed into the Petri dish. The aggression score of each interacting pair was recorded every minute for five minutes, and the number of dead ants of each color was recorded at five minutes, one hour, and two hours. As a control, the average mortality of un-powdered crazy ants kept in a Petri dish for two hours was tested.

Because data were non-normal, even with transformations, a Wilcoxon signed rank test was used to determine if peak interaction score varied significantly when colony pairs differed by prey type, sugar level, both, or neither. The same test was also used to determine if diet differences affected mortality after one and two hours. Additionally, a Wilcoxon signed rank test was used to compare one and two hour mortality of all pairings of different nests. It was also used to compare powdered controls of ten workers from the same colony and un-manipulated controls of five workers from the same colony.

### Crazy Ants Receiving Different Diets vs. Fire Ants

In order to test the effect of crazy ant diet on interactions with fire ants, aggression assays were again performed on July 31, 2008, using five crazy ant workers and five small fire ant workers. Each crazy ant colony was used only once. Two fire ant nests were used twice, but each colony pair was unique. There were six aggression assays performed for each of the four treatments for a total of 24 colony pairings. The aggression scores were recorded every minute for five minutes, and mortality was recorded at five minutes, one hour, and two hours.

To determine the effect of diet on aggression and mortality of the two species, two-way ANOVA’s were performed with prey type, sugar level, and the interaction of prey type and sugar level as factors. The following response variables had a normal distribution and therefore were tested using the ANOVA described above: the average interaction score across all time periods, the average number of crazy ants in engaged in fights, the average number of fire ants involved in fights, and crazy ant mortality after two hours. The mortality of fire ants after two hours was square root transformed prior to testing for a diet effect with an ANOVA. Mortality after two hours was used because it was greater and had a more normal distribution than one-hour mortality, and in only two cases were all five of the ants from a colony in a Petri dish dead (both occasions were fire ants). In order to test for a difference between crazy ants and fire ants in average number of workers fighting and mortality after two hours, the fire ant values were subtracted from the crazy ant values for each response variable. A Wilcoxon signed rank test was used to test if the mean of the difference in numbers of workers fighting was significantly different from zero, and an ANOVA was used to determine if the mean of the difference in mortality after two hours was affected by sugar level, prey type, or an interaction of the two variables. Also, a Wilcoxon signed tank test was used to see if prey or sugar level significantly affected the difference between the average number of crazy ant and fire ant workers engaged in fights.

### Crazy Ants vs. Small and Large Fire Ants

Aggression assays between crazy ant workers and fire ant workers of different sizes were conducted on July 16, 2008. Each of 12 fire ant nests was used twice as a source of workers, the first time with five small workers paired up with five crazy ant workers, and the second time with five large workers paired up with five crazy ant workers from a different colony, creating a total of 24 aggression trials. Aggression scores and number of ants fighting were recorded every minute for ten minutes. Mortality was recorded after ten minutes, and for ten pairings, mortality was also recorded at one hour. In order to quantify the size difference between crazy ants and small and large fire ant workers, the head length (from the front of the clypeus to the posterior margin of the head) of ten ants from each of the three groups was measured. Head length is considered the most reliable predictor of body size across ant species [31]. The average and standard error of the length was calculated for each group, and the head lengths were compared using a one-way ANOVA, followed by a post hoc Tukey’s test (Table 1).

**Table 1:** Head lengths of fire ants and crazy ants and fire ant colony size distribution.

Ant	Length (mm)	Level	Average number	Percent of Sample
Large fire	1.28±0.02	A	4.73±5.02	16.1%
Medium fire	N/A	N/A	10.91±7.46	37.2%
Small fire	0.78±0.02	B	12.72±5.48	46.7%
Crazy	0.69±0.01	C	N/A	N/A

Ants that do not share the same level letter significantly differ in head length (as indicated in a Tukey’s post hoc test). Average number is the number of fire ants in each size class in a haphazardly selected sample of 21 to 36 ants from ten nests. Percentages are from an average sample size of 28.36 ants. Errors are ±1 SE.

Overall average aggression score, average number of fire ants fighting, and average number of crazy ants fighting all met assumptions of normality and therefore were analyzed using a one-way ANOVA to determine the effect of fire ant size on the response variables. Average mortality after ten minutes was minimal and was often zero, thus it was not used in an analysis. Mortality for both crazy ants and fire ants after one hour was non-normal and could not be transformed to achieve normality and therefore was analyzed using a Wilcoxon signed rank test. To determine if the average number of ants fighting or the number of dead ants after one hour differed by species, Wilcoxon signed rank tests were performed to test whether the mean of the fire ant values subtracted from the crazy ant values for each response variable was significantly different from zero. The same metrics were also tested for a significant effect of fire ant size using Wilcoxon signed rank tests. Additionally, average crazy ant and fire ant mortality after one hour was compared to survival of control Petri dishes containing either five crazy ants or five fire ants using Wilcoxon signed rank tests on the fire ant mortality and the square root of crazy ant mortality after one hour. We used P<0.05 to denote significance and 0.05≤P<0.10 to denote a trend. All data are available from the authors by request.

## Results

### Crazy Ants Receiving Different Diets

There was no fighting between crazy ants in any of the aggression assays; the highest aggression score was a 2, which occurred only five times during the 144 observation periods. Peak interaction score therefore was not significantly affected by differences in diet (Z = 1.57, p = 0.667). There was no mortality for any of the ants after five minutes. Neither mortality after one hour (Z = 2.95, p = 0.340) or mortality after two hours (Z = 2.42, p = 0.491) were significantly affected by diet differences. The overall average mortality after two hours was 1.09±0.21 out of five ants. This was not significantly different from the average two-hour mortality of the five sets of un-powdered ants in a Petri dish (Z = 0.15, p = 0.694). Additionally, the four pairs that received the same diet did not have significantly different mortality than the five un-powdered controls (Z = 0.00, p = 1.000).

### Crazy Ants Receiving Different Diets vs. Fire Ants

**Figure 1:**
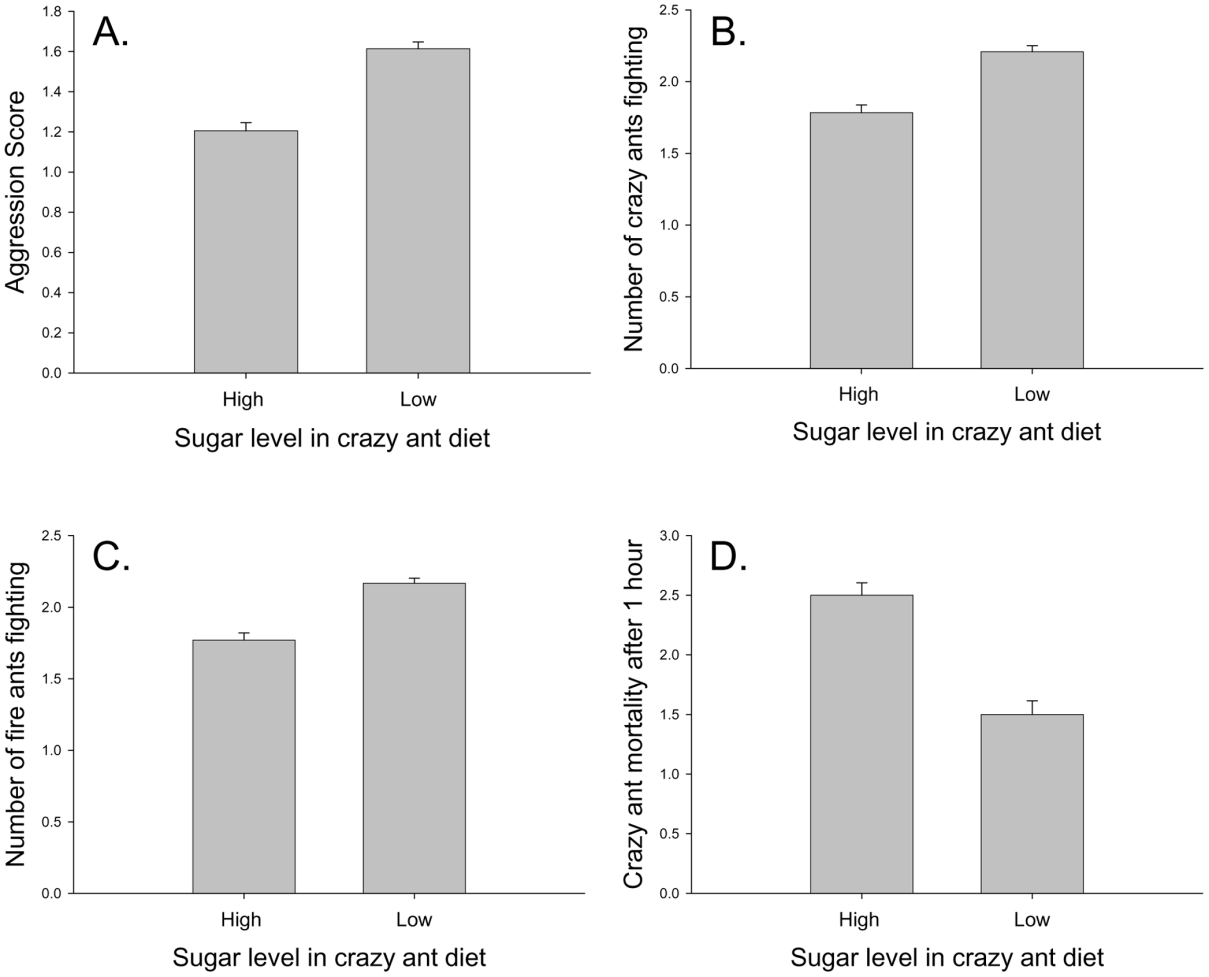
Crazy ants receiving different diets vs. fire ants. The effects of sugar level in crazy ant diet on: A) Average aggression score for each set of fire ants and crazy ants. Aggression scores are assigned according to the scale described by Suarez et al. [30]. Aggression scores for interactions between fire ants and crazy ants were higher when crazy ants received a low sugar diet. B) Average number of crazy ants fighting during each observation (out of a total of five ants). On average, crazy ants receiving a low sugar diet were more likely to engage in fights with fire ants. C) Average number of fire ants fighting during each observation (out of a total of five ants). Fire ants were more likely to be engaged in fights with crazy ants on a low sugar diet. D) Crazy ant mortality after two hours. On average, crazy ant mortality was higher for ants receiving a high sugar diet. Mortality counts are out of a possible mortality of five ants. Means +1 SE.

Sugar level significantly affected the average interaction score (F_1,20_ = 4.97, p = 0.037), though prey type had no effect (F_1,20_ = 0.75, p = 0.398) and the interaction of the two factors was not significant (F_1,20_ = 1.16, p = 0.294). Ants receiving a lower sugar diet were more aggressive when interacting with fire ants than did crazy ants that consumed high sugar diets (Fig. 1A). There was also a trend for replicates of lower sugar diet treatments to have a higher average number of both crazy ants (F_1,20_ = 3.16, p = 0.091, Fig. 1B) and fire ants (F_1,20_ = 3.28, p = 0.085, Fig. 1C) involved in fights. Prey type and the interaction of the two resources were not significant factors in either the number of crazy ants fighting (F_1,20_ = 0.10, p = 0.757 for prey; F_1,20_ = 1.27, p = 0.2730 for the interaction) or the number of fire ants fighting (F_1,20_ = 0.22 and p = 0.644 for prey, F_1,20_ = 0.95 and p = 0.341 for the interaction). After two hours, there was a trend for crazy ants that consumed high sugar diets to have more mortality than crazy ants fed a low sugar diet (F_1,20_ = 3.19, p = 0.090, Fig. 1D). Neither prey type (F_1,20_ = 0.09, p = 0.769) nor the interaction of sugar and prey (F_1,20_ = 0.09, p = 0.769) significantly affected crazy ant mortality, and none of the response variables affected fire ant mortality (F_1,20_ = 0.21, p = 0.651 for sugar level; F_1,20_ = 2.84, p = 0.108 for prey type; F_1,20_ = 0.59, p = 0.453 for the interaction).

### Crazy Ants vs. Small and Large Fire Ants

**Figure 2:**
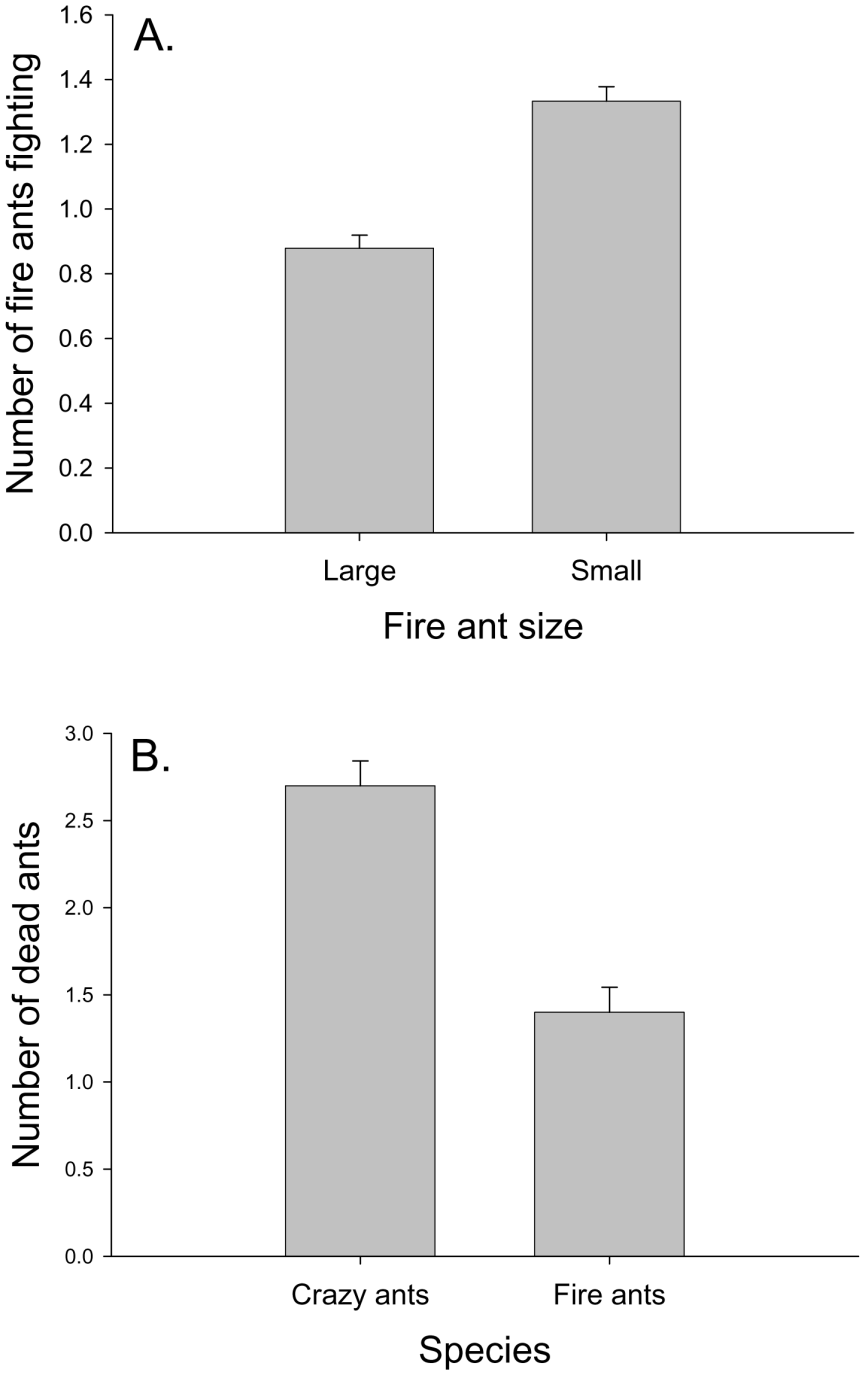
Crazy ants vs. small and large fire ants. A) The effect of fire ant worker size on the average number of fire ants fighting during each observation (out of a total of five fire ants). Small fire ants fought more often than large fire ants. B) The number of dead crazy ants and dead fire ants after one hour. Crazy ants had higher mortality than fire ants. The maximum possible mortality was five ants. Means +1 SE.

There was no effect of fire ant worker size category on overall average aggression score (F_1,22_ = 1.20, p = 0.285). The average aggression score of replicates with large fire ants was 1.00±0.11, and the average aggression score of dishes with small fire ants was 1.18±0.11. There was no effect of fire ant size on the average number of crazy ants fighting during a trial (F_1,22_ = 2.91, p = 0.102), but there was a significant effect of fire ant size on the average number of fire ants fighting (F_1,22_ = 4.76, p = 0.040), with small fire ants having an average of 51.6% more workers fighting than large fire ants fighting during a given observation period (Fig. 2A). Fire ant size category had no effect on either crazy ant mortality (Z = 0.87, p = 0.386) or fire ant mortality (Z = 1.20, p = 0.230) after one hour.

Comparing the response variables of the two species, there was a trend for crazy ants to have both a higher average number of workers engaged in fights (Z = 12.00, p = 0.063) and higher mortality after one hour (Z = 13.50, p = 0.063, Fig. 2B). On average, 1.16±0.12 crazy ant workers were fighting during each observation period, while 1.11±0.11 fire ant workers were engaged in fights. Crazy ant mortality when fighting fire ants was significantly higher than that of control crazy ants (Z = −2.65, p = 0.008). Controls averaged 0.2±0.2 dead crazy ants after one hour, whereas crazy ants fighting fire ants had an average mortality of 2.7±0.45 ants after one hour. Likewise, fighting fire ants had significantly higher mortality after one hour than controls (Z = −2.01, p = 0.037).

## Discussion

Diet significantly affected interspecific interactions between crazy ants and fire ants. Crazy ants receiving a lower sugar diet were more aggressive and tended to fight more with fire ants than crazy ants in the high sugar treatment (Fig. 1A–C). On the other hand, there was a trend for crazy ants on the elevated sugar diet to experience more mortality after one hour than their low sugar counterparts (Fig. 1D), though sugar level had no effect on fire ant mortality. Prey type had no effect on antagonistic interactions, which is consistent with the findings of Alloway et al. [32], who found no difference in aggression between two species of Leptothoracine ants that were fed diets that differed in protein type and vitamin and mineral source but not sugar source. Overall, the two species did not differ significantly in the average number of workers fighting or in mortality after one hour, and there was no effect of diet on the difference between the two species.

Carbohydrates play an important role in ant colony growth and adult activity levels [19], [33]. Therefore, it is expected that sugar level would affect interspecific antagonistic interactions. We found evidence that crazy ants fed a low carbohydrate diet were more aggressive than those fed high carbohydrate diets (Figs. 1AB). This is the opposite pattern found in other studies of the effects of carbohydrates on aggression in ants; ant workers are typically more aggressive when fed high sugar diets [19], [16]. Furthermore, per capita activity (based on the exploration of a structure not containing food) was higher in the low sucrose treatment than in either the sucrose-free treatment or the high sucrose treatment. In light of these findings, it is conceivable that crazy ants that are limited by sugar may experience increased per capita activity and therefore engage in more frequent and aggressive interactions with another species, in this case fire ants. In sum, the findings of increased aggression and decreased mortality on a low sugar diet provide surprising new insight in the behavior of crazy ants and may have important implications in the success and spread of the crazy ant.

Diet differences had no effect on intraspecific crazy ant interactions after 12 days. No fighting was observed between any of the workers, and the most aggressive interaction recorded was avoidance, which occurred only five times. Mortality did not differ among pairs that received different diets, pairs receiving the same diet, and controls of five ants. The lack of aggression between crazy ants that received different diets may indicate that diet changes alone do not disrupt the chemical profile of the workers enough to overcome the likely unicolonial nature of the introduced population. Genetic effects may also be important for nestmate recognition [34]. However, several studies that have examined the effect of diet on intraspecific aggression in other ants have found that aggression can be induced [17], [14], [18], [15]. For example, Corin et al. [15] found aggression between unicolonial Argentine ant nests after 56 days of diet manipulation, and Silverman and Liang [14], found that former nestmates behaved aggressively towards one another after 28 days on different diets. Additionally, Lim et al. examined interactions between nestmates of *Paratrechina longicornis* and found antagonistic behavior beginning at 21 days after the implementation of diet treatments [35]. Together, these studies suggest that the absence of aggression in Caribbean crazy ants may be due to the treatments not being maintained long enough for aggressive interactions to develop. Nevertheless, one study of Argentine ants showed antagonistic behavior between nestmates after as little as two minutes of contact with a prey item, the brown-banded cockroach, *Supella longipalpa* [17]. Therefore, changes in nestmate recognition and aggression are therefore conceivable within 12 days, and the prey types used in the diets, though from different orders of insects, may not have had distinct enough chemical profiles to affect nestmate recognition.

The results of the trials between crazy ants and fire ants of different sizes suggested that more crazy ants than fire ants were involved in fights and that crazy ants suffered more mortality than fire ants after one hour (Fig. 2B). Nevertheless, both ant species experienced higher mortality after exposure to the other species than either species experienced in isolation. Fire ant worker size had little affect on aggressive interactions with crazy ants, as aggression score, the number of crazy ants fighting, and mortality for both species were not influenced by fire ant size. The exception is that small fire ants fought more than 50% more often than large fire ants (Fig. 2A). The tendency of small fire ant workers to fight more often may be biologically important, as small fire ants are nearly three times more common than large fire ants based on the average distribution of worker sizes in our field-collected nests. This means that crazy ants are more likely to encounter aggressive small workers than large workers that avoid fights. The increase in fighting does not correlate with an increase in mortality for either crazy ants or fire ants, however, suggesting that the heightened aggression may not be important for colony population dynamics. Overall, crazy ant workers were twice as likely to die when fighting fire ants as fire ant workers were in those encounters (Fig. 2B). Fire ants are equipped with a stinger whereas crazy ants spray formic acid via an acidopore. It is possible that the ability to sting makes fire ants a more potent combatant than crazy ants.

The findings of this study give insights on the biotic factors affecting the spread of introduced Caribbean crazy ant populations. The most common ant in the introduced range, the red imported fire ant, has less mortality when fighting crazy ants. Additionally, small fire ants, which are most common, engage in fights more often than larger fire ants. Together, these results suggest that fire ants may have a competitive advantage over crazy ants and may be able to defend their territory from a neighboring colony of crazy ants. On the other hand, when crazy ants consume a reduced sugar diet, they become more aggressive, they engage in fights more often, and they have less mortality following battles with fire ants. Therefore, a crazy ant colony that has been competitively excluded from sugar sources by fire ants or other ants may have a better chance of overpowering fire ants to gain access to resources. These possibilities should be explored in field experiments. In sum, these findings may help explain the occurrence of large supercolonies of crazy ants in habitats where competition with fire ants is expected to be high and may help predict the future invasive spread of crazy ants.

## References

[1] Mack RN, Simberloff D, Lonsdale WM, Evans H, Clout M, et al (2000) Biotic invasions: Causes, epidemiology, global consequences, and control. Ecological Applications 10: 689–710.

[2] Mitchell CE, Power AG (2003) Release of invasive plants from fungal and viral pathogens. Nature 421: 625–627. 12571594 10.1038/nature01317

[3] Ness JH, Bronstein IL (2004) The effects of invasive ants on prospective ant mutualists. Biological Invasions 6: 445–461.

[4] Torchin ME, Mitchell CE (2004) Parasites, pathogens, and invasions by plants and animals. Frontiers in Ecology and the Environment 2: 183–190.

[5] Drescher J, Feldhaar H, Bluethgen N (2011) Interspecific aggression and resource monopolization of the invasive ant *Anoplolepis gracilipes* in Malaysian Borneo. Biotropica 43: 93–99.

[6] Savage AM, Johnson SD, Whitney KD, Rudgers JA (2011) Do invasive ants respond more strongly to carbohydrate availability than co-occurring non-invasive ants? A test along an active *Anoplolepis gracilipes* invasion front. Austral Ecology 36: 310–319.

[7] Wundrow EJ, Carrillo J, Gabler CA, Horn KC, Siemann E (2012) Facilitation and competition among invasive plants: a field experiment with alligatorweed and water hyacinth. PloS One 7: e48444. 23119021 10.1371/journal.pone.0048444 PMC3484115

[8] Walters AC, Mackay DA (2005) Importance of large colony size for successful invasion by Argentine ants (Hymenoptera : Formicidae): Evidence for biotic resistance by native ants. Austral Ecology 30: 395–406.

[9] Menke SB, Fisher RN, Jetz W, Holway DA (2007) Biotic and abiotic controls of argentine ant invasion success at local and landscape scales. Ecology 88: 3164–3173. 18229850 10.1890/07-0122.1

[10] Ricklefs RE (2001) The economy of nature. New York, NY: W. H. Freeman and Company. 577 p.

[11] Savolainen R, Vepsalainen K (1988) A competition hierarchy among boreal ants - impact on resource partitioning and community structure. Oikos 51: 135–155.

[12] Hölldobler B, Wilson EO (1990) The Ants. Berlin: Springer.

[13] Andersen AN, Patel AD (1994) Meat ants as dominant members of Australian ant communities - an experimental test of their influence on the foraging success and forager abundance of other species. Oecologia 98: 15–24. 28312791 10.1007/BF00326085

[14] Silverman J, Liang D (2001) Colony disassociation following diet partitioning in a unicolonial ant. Naturwissenschaften 88: 73–77. 11320891 10.1007/s001140000198

[15] Corin SE, Abbott KL, Ritchie PA, Lester PJ (2007) Large scale unicoloniality: the population and colony structure of the invasive Argentine ant (*Linepithema humile*) in New Zealand. Insectes Sociaux 54: 275–282.

[16] Sorvari J, Theodora P, Turillazzi S, Hakkarainen H, Sundstrom L (2008) Food resources, chemical signaling, and nest mate recognition in the ant *Formica aquilonia* . Behavioral Ecology 19: 441–447.

[17] Liang D, Blomquist G, Silverman J (2001) Hydrocarbon-released nestmate aggression in the Argentine ant, Linepithema humile, following encounters with insect prey. Comparative Biochemistry and Physiology, Part B 129: 871–882. 10.1016/s1096-4959(01)00404-3 11435142

[18] Buczkowski G, Silverman J (2006) Geographical variation in Argentine ant aggression behaviour mediated by environmentally derived nestmate recognition cues. Animal Behaviour 71: 327–335.

[19] Grover CD, Kay AD, Monson JA, Marsh TC, Holway DA (2007) Linking nutrition and behavioural dominance: carbohydrate scarcity limits aggression and activity in Argentine ants. Proceedings of the Royal Society B-Biological Sciences 274: 2951–2957. 10.1098/rspb.2007.1065 PMC2291163 17878138

[20] Holway DA, Lach L, Suarez AV, Tsutsui ND, Case TJ (2002) The causes and consequences of ant invasions. Annual Review of Ecology and Systematics 33: 181–233.

[21] Meyers JM (2008) Identification, distribution and control of an invasive pest ant, *Paratrechina* sp. (Hymenoptera, Formicidae), in Texas. PhD thesis. College Station, TX: TX A&M University.

[22] Trager JC (1984) A revision of the genus *Paratrechina* (Hymenoptera: Formicidae) of the continental United States. Sociobiology 9: 49–162.

[23] Zhao LM, Chen J, Jones WA, Oi DH, Drees BM (2012) Molecular comparisons suggest Caribbean crazy ant from Florida and Rasberry crazy ant from Texas (Hymenoptera: Formicidae: *Nylanderia*) are the same species. Environmental Entomology 41: 1008–1018.

[24] Gotzek D, Brady SnG, Kallal RJ, LaPolla JS (2012) The importance of using multiple approaches for identifying emerging invasive species: the case of the Rasberry crazy ant in the United States. PLoS ONE 7: e45314. 23056657 10.1371/journal.pone.0045314 PMC3462614

[25] Lowe S, Browne M, Boudjelas S, DePoorter M (2004) 100 of the world’s worst invasive species: a selection from the Global Invasive Species Database. Invasive Species Specialist Group, Gland, Switzerland.

[26] Buren WF, Allen GE, Whitcomb WH, Lennartz FE, Williams RN (1974) Zoogeography of imported fire ants. Journal of the New York Entomological Society 82: 113–124.

[27] Williams DF (1994) Control of the introduced pest *Solenopsis invicta* in the United States. In: Williams DF, editor. Exotic ants: biology, impact, and control of introduced species. Boulder, CO: Westview Press.

[28] Pimentel D, Lach L, Zuniga R, Morrison D (2000) Environmental and economic costs of nonindigenous species in the United States. Bioscience 50: 53–65.

[29] Roulston TH, Buczkowski G, Silverman J (2003) Nestmate discrimination in ants: effect of bioassay on aggressive behavior. Insectes Sociaux 50: 151–159.

[30] Suarez AV, Tsutsui ND, Holway DA, Case TJ (1999) Behavioral and genetic differentiation between native and introduced populations of the Argentine ant. Biological Invasions 1: 43–53.

[31] Kaspari M, Weiser MD (1999) The size-grain hypothesis and interspecific scaling in ants. Functional Ecology 13: 530–538.

[32] Alloway TM, Leighl A, Ryckman D (1991) Diet does not affect intercolonial fighting in Leptothoracine ants. Insectes Sociaux 38: 189–193.

[33] Helms KR, Vinson SB (2008) Plant resources and colony growth in an invasive ant: The importance of honeydew-producing Hemiptera in carbohydrate transfer across trophic levels. Environmental Entomology 37: 487–493. 18419921 10.1603/0046-225x(2008)37[487:pracgi]2.0.co;2

[34] Gotzek D, Ross KG (2009) Current status of a model system: the gene Gp-9 and Its association with social organization in fire ants. PLoS ONE 4: e7713. 19893635 10.1371/journal.pone.0007713 PMC2767508

[35] Lim SP, Chong ASC, Lee CY (2003) Nestmate recognition and intercolonial aggression in the crazy ant, *Paratrechina longicornis* (Hymenoptera : Formicidae). Sociobiology 41: 295–305.

